# An Exploration of Current and Perspective Semen Analysis and Sperm Selection for Livestock Artificial Insemination

**DOI:** 10.3390/ani11123563

**Published:** 2021-12-15

**Authors:** Dalen Zuidema, Karl Kerns, Peter Sutovsky

**Affiliations:** 1 Division of Animal Sciences, University of Missouri, Columbia, MO 65211, USA; dmzfdb@umsystem.edu (D.Z.); kkerns@iastate.edu (K.K.); 2 Department of Animal Science, Iowa State University, Ames, IA 50011, USA; 3 Department of Obstetrics, Gynecology and Women’s Health, University of Missouri, Columbia, MO 65211, USA

**Keywords:** artificial insemination, sperm sexing, livestock andrology, semen analysis, sperm selecting, semen storage

## Abstract

**Simple Summary:**

Artificial insemination is a crucial technology for livestock producers world-wide. This technology has afforded producers access to premier genetics without having to physically own superior sires. The improvement of this technology can have a large impact on the livestock industry, especially those sectors of the industry which rely heavily on artificial insemination. This article will review ongoing improvements being made to artificial insemination and their potential impacts.

**Abstract:**

Artificial insemination of livestock has been a staple technology for producers worldwide for over sixty years. This reproductive technology has allowed for the rapid improvement of livestock genetics, most notably in dairy cattle and pigs. This field has experienced continuous improvements over the last six decades. Though much work has been carried out to improve the efficiency of AI, there are still many areas which continue to experience improvement, including semen analysis procedures, sperm selection techniques, sperm sexing technologies, and semen storage methods. Additionally, the use of AI continues to grow in beef cattle, horses, and small ruminants as the technology continues to become more efficient and yield higher pregnancy rates. In this review, AI trends in the various livestock species as well as cutting edge improvements in the aforementioned areas will be discussed at length. Future work will continue to refine the protocols which are used for AI and continue to increase pregnancy rates within all livestock species.

## 1. Introduction

The world-wide consumption of animal products has grown by over 20% in the past ten years [1]. The increase in meat and dairy consumption is expected to rise worldwide, with the majority of growth taking place in developing countries [1]. The rise in demand will be met by applying cutting-edge technology to agricultural production and by improving on the current best practices; artificial insemination (AI) is one such technology in need of improvement. When used by the livestock industry, AI can improve both quality and quantity of animal products.

AI has been commercially available to livestock producers for over sixty years [2]. The primary benefit of utilizing AI is the ability to rapidly improve the genetic quality of a herd using a premier male animal’s genetics without a producer having to purchase that specific male. Rather, a producer can buy semen doses from several premier sires with valuable genomes and production traits to improve their herd’s genetic and phenotypic profile in an affordable manner without having to buy or house the sires. AI is used to increase herd productivity by using sires that provide superior production traits to their offspring. Other benefits of AI are the reduced risk of disease transmission between animals, and reduced risk of other animals and workers being injured by male animals. AI maximizes the use of a genetically superior males, enabling them to sire hundreds or thousands of offspring by extending and shipping their semen for wider use. For example, prior to the use of AI, one mature bull could be used to breed approximately 60 cows in a 70 day breeding season each year [3]. The adoption of AI has allowed us to breed 100 cows or more from a single ejaculation [4] and in an extreme example has allowed one bull to father over 500,000 offspring [5]. Though this is a dramatic example of the potential impact one sire can have, using AI, maximization of individual males albeit to a lesser extent, exist in other species as well.

This technology was first commercialized in the dairy industry in the 1930s and 1940s [6]. Since then, it has been adopted for use in all major livestock species. The world-wide AI industry is currently valued at USD 3.95 billion and expected to continue to grow [7]. This increase will result from a growth in popularity among producers and improvements in semen diagnostics, handling, storage, as well as breeding protocols, and sire selection. Though not specifically addressed in this paper, the authors recognize that poultry and cervids also make up portions of the livestock AI industry. These species have their own list of accomplishments and challenges, many of which may overlap with those species which are covered within the scope of this paper.

In this paper, semen analysis, sperm selection, semen storage, and their potential impact on the AI industry will be discussed. The basic process of AI is the same across species; collected semen is used to manually breed an animal. However, there are differences between species, including varying adoption levels of AI within a species, the semen storage methods used, and the tolerance of spermatozoa to processing. Some of these species’ differences will be addressed below and followed by a discussion of state-of-the-art improvements to the industry.

### 1.1. Cattle

As of 2019, the cattle AI industry accounted for 46.5% of the market value associated with AI [7]. Cattle AI continues to grow in popularity. In the U.S., 60% of dairy operations use AI [7]. Astoundingly different, only about 8% of U.S. beef producers use AI [8]. The popularity of AI in the dairy industry is not exclusive to the U.S. In Northern Europe, Israel, Japan and New Zealand, AI accounts for 80 to 90% of all breedings in dairy cattle. However, this does not hold true for all countries. There are still many countries in which the majority of dairy producers still utilize natural service, and this is especially true in developing countries [9,10]. In general, cattle AI technology is more advanced than that in the other livestock species. Frozen semen is almost exclusively used for cattle AI in the U.S., which has been advantageous for shipping and storing semen [11]. This is quite different from the other livestock species in which frozen semen makes up a very low percentage of AIs conducted. A large incentive exists to mimic the cattle industry by adapting semen freezing practices in other species [12]. Unfortunately, attempts to commercialize semen freezing in other species have yet to be adopted on a large scale due to its inefficiency. It should be noted that in some countries, such as New Zealand and Ireland, cooled liquid semen is commonly used for cattle AI. There are several reasons for this practice. Bovine liquid semen doses require as little as one tenth the spermatozoa numbers when compared with frozen doses, liquid semen has generally higher fertility than frozen semen as well, and these countries subscribe to seasonal dairy production systems. Liquid semen can utilize fewer spermatozoa and maintain higher levels of fertility because the process of freezing spermatozoa decreases sperm viability [13]. This will be discussed further in the paragraph “Semen Storage”. Although sex-sorted then cryopreserved spermatozoa can utilize similarly reduced numbers of cells, these sex-sorted cells include dead spermatozoa (as defined by inclusion of food coloring dye) discarded during the sorting process. Further, a sex-sorted bull typically has to be proven to have good fertility before being a candidate for sorting. Seasonal dairy production requires that a large amount of semen doses be available for producers during the exact same time of year. This means each ejaculate must be maximized and producers want as high pregnancy rates as possible during the breeding season. Thus, liquid semen works well for producers in these countries. Producers that utilize seasonal dairy production systems routinely choose liquid semen as opposed to frozen semen because it best fits their reproduction goals and their management styles [10].

Finally, sexed semen technology has been commercialized in the cattle industry [14]. This technology has actively been attempted in the other livestock species [15,16,17] and is on the cusp of being commercialized for both pigs and small ruminants. Cattle AI can still benefit greatly from further improvements but is leading the AI field.

### 1.2. Swine

Despite cattle making up the largest portion of the AI industry in value [7], the U.S. swine industry is close behind and actually conducts the highest percentage of AI out of any livestock species, with more than 95% of all pig litters in the U.S. being sired through AI [18]. Different from cattle, nearly 99% of all U.S. pig AIs are conducted using liquid stored semen rather than frozen [19]. Liquid extenders have seen a great deal of innovation through the years [13]; however, the shelf life of these liquid extenders is an area which continues to receive attention. Cryopreservation would further extend the shelf life of boar semen and has been researched extensively, yet semen freezing in pigs is not efficient enough for wide-spread use and only accounts for about 1% of AI service conducted [13]. Consequently, frozen semen is mostly used for (1) backing up valuable genetics in the face of disease threats; (2) backing up genetics in the event genetic selection takes an undesired route or if phenotypical traits desired change with the economy (e.g., genetic lines that are more feed efficient versus faster growing); and (3) importing new genetic lines across country borders where regulations do not allow for timely use and temperature shipping conditions of fresh semen.

Within the swine industry, it is common practice to pool boar semen to compensate for a boar’s potential subpar fertility for biological insurance. This practice allows sub-fertile boars to continue to perpetually exist within the population. Further, there are concerns that some sub-fertile boars can bring down the average pregnancy rate and litter sizes. A greater focus on boar genomics and biomarker-based semen quality traits would reduce the need for and use of pooling by improving boar fertility. The increased emphasis on evaluating male fertility through genomics and semen quality measurements can be applied to and boost productivity of AI in all livestock species.

Finally, sperm sexing is a technology of great interest to the swine industry as well. Selecting for female offspring through sexing would save producers from dedicating resources for castration and greatly benefit the multiplier farms that produce replacement gilts [20]. Sexing Technologies, the company that first commercialized the USDA ARS invention into sexed cattle semen, recently purchased a swine genetics company called Fast Genetics. Through this acquisition, Sexing Technologies is attempting to commercialize swine-sexed semen for eventual widespread use [21].

### 1.3. Horses

Equine AI is much less popular than swine or cattle AI; in fact, several horse registries are actively opposing equine AI and hindering its expansion by excluding AI-derived horses from registering. The most impactful example of this is The American Jockey Club, which is responsible for the registry of racing thoroughbreds. For a horse to become registered in this club, it must be conceived via natural breeding. AI, cloning, and embryo transfer are all prohibited assisted reproductive technology (ART) practices for horses registered to this prolific club [22]. Despite the lack of popularity and outright rejection of AI by some in the horse industry, horse AI has continued to grow over the past twenty-five years [11]. Most horse AIs are currently conducted using cooled liquid extenders, similar to swine extenders. Horse semen is routinely stored at regular refrigeration temperature (4 °C) compared with swine semen (17 °C), but must be used within 24 h after which the quality of the spermatozoa is greatly diminished [11]. The swine industry is starting to adapt to this hypothermic storage application in the face of eliminating antibiotics with long term semen storage metrics remaining acceptable well beyond 24 h [23].

Stallions are frequently selected based on performance in competitions without emphasis on fertility or semen quality. Because of this, a great variability in semen quality exists between stallions. This explains the variable pregnancy rates associated with equine AI [11]. Equine AI is another AI industry which would benefit from an increased emphasis on male fertility and semen quality. Despite the equine sector of the AI industry having such limitations, equine AI is anticipated to continue growing over the next seven years as the efficiency grows and producers gain more interest [7].

### 1.4. Small Ruminants

Sheep and goat AI have received substantially less attention than both swine and cattle AI. Additionally, AI in both of these species is more complicated due to the size of the animals and the tortuous anatomy of the cervix in sheep [11]. Sheep AI is often conducted using laparoscopic AI, a method which bypasses the cervix but requires surgery to be conducted on the animal which is being bred. This surgery raises the costs of breeding, as well as the stress on the animal but is a method which results in successful pregnancies [24]. Currently, the majority of goat and sheep AI is conducted using liquid, extended semen, similar to the equine and swine AI [11]. There is a desire to increase the use and viability of frozen semen doses for small ruminants. However, as in other species, there is currently an association with frozen semen and lower fertility rates in small ruminants even when laparoscopic AI is employed [25]. Sexed semen is beginning to be commercialized for small ruminants for the first time due to a collaborative effort between Animal Breeding Europe and Cogent that began in August of 2020 [26]. These companies will provide the first widespread sexed sheep semen products. Sheep AI specifically is benefitting from the introduction of new catheters and breeding protocols which are able to reliably pass semen through the sheep cervix, eliminating the need for laparoscopic AI, and thus increasing the feasibility and subsequently the popularity of sheep AI [11,27].

## 2. Improving AI Technology

Despite its massive contributions to livestock production, there are numerous ways in which AI can be improved. These improvements are driven by new technologies and deeper understanding of reproductive physiology. The AI industry has many promising technologies which are being investigated and adopted for use. These technologies utilize new paradigms related to reproductive physiology to drive improved AI outcomes. Such technologies have been applied to semen analysis, sperm selection, and semen storage.

Improving semen quality control for use in AI has been studied using many different approaches. The conventional method for semen quality analysis is the use of a light microscope to evaluate sperm count, motility, and morphology. The use of subjective motility, and morphology as selection criteria is somewhat reliable but is associated with variable fertility rates, especially in horses [28]. Analyzing semen using more accurate and objective sperm quality parameters will improve sire AI fertility [29]. Semen analysis with such parameters can be applied as selection criteria for sires and used to eliminate those sires which lack an objective threshold of fertilization potential. Objective, biomarker-based semen analysis can also be used to evaluate samples of individual semen collections to ensure the quality of each ejaculation in sires with acceptable, but varied semen quality and AI fertility.

Finally, semen analysis parameters can be used in conjunction with semen purification methods during processing to improve the quality of sperm within each dose of semen, particularly when processing semen of high value sires with superior genetic traits. Additionally, using sperm quality parameters that are correlated with better storability and higher tolerance to cryopreservation will improve semen shelf-life, further benefitting the AI industry [30]. All these improvements can be achieved using cutting-edge semen analysis and sperm selection methods.

## 3. Semen Analysis

Two standout technologies have been developed to accurately and objectively analyze sperm quality. Both implement computer software which can calculate sperm quality based on objective measurements. Computer-assisted semen analysis (CASA) is a system which analyzes sperm count (thus concentration), morphology, and motility objectively and accurately [31]. CASA evaluates these quality measures more accurately than conventional light microscopy and has been enhanced with the ability to analyze sperm viability and DNA fragmentation, which are markers of sperm quality; these advancements will further improve the utility of the CASA system [32].

The second system being developed is even more powerful than CASA in terms of its throughput and ability to analyze semen quality based on objective, machine-measurable parameters. Flow cytometry (FC) based semen analysis is an approach that works with specific probes to evaluate sperm viability, DNA content, acrosomal integrity, mitochondrial membrane potential, and various determinants of sperm quality including surface proteins, glycans, and select ions [33,34] linked to sperm function and quality [35,36,37]. This approach is termed biomarker-based sperm analysis [30]. Fluorescent probes are used to detect specific sperm quality biomarkers. These probes induce a level of fluorescence to be emitted by every single spermatozoon positive for the biomarker used and the emission is measured by a light detector in the flow cytometer and used to evaluate the quality of the entire semen sample [32]. This technology can be further refined to sort spermatozoa based on the different fluorescent levels emitted by each spermatozoon, which is how sexed semen is currently sorted based on quantification of the fluorescence emitted by a DNA stain. Similar to sperm sexing, the biomarker-based fluorescence activated cell sorting would require a modified cell sorter to sort the spermatozoa within a semen sample for sperm quality characteristics. Such an approach may be accelerated by the development of so-called gentle, low-pressure cell sorters currently introduced to market.

Currently, CASA is the simpler and more affordable option, that allows quick and repeatable sperm motility and morphometry assessment. FC-based semen analysis adds additional analysis power because identifying specific biomarkers provides a more in-depth understanding of the sperm quality of individual sires and ejaculates. Despite the greater cost, FC-based semen analysis has begun to be adopted within the cattle AI industry [38,39,40]. As flow cytometry continues to grow in popularity and the technology is further developed, these systems will become more affordable and be further adopted for use in the AI industry at large, accelerating a trend that started a decade ago. This biomarker-based flow cytometric semen analysis allows for the detection of subtle differences in sperm quality that cannot be measured by CASA alone. As the AI industries begin to adopt more objective semen analysis tool such as CASA, the next step will be to include biomarker-based analysis in order to further ensure best possible semen quality. In terms of the swine industry specifically, the use of biomarker-based semen analysis will help reduce the use of semen pooling practices and reserve this practice only when necessary. Thus, males with inferior semen quality can more quickly be removed from the breeding herd. We acknowledge pooling might not be completely eliminated in the swine industry because these analyses are impractical in some settings. Additionally, swine producers are more concerned about genetic lines and the herd at large than the individual animals. This is different than how the other livestock industries are currently managed because of the differences in genetic seedstock and commodity production flow. However, even with this caveat, ensuring that the boar herd uses males with the best semen quality possible based on objective measurements including both CASA and biomarker-based analysis will help the level of boar fertility achieved even when pooled and can be pushed to a higher quality with these tools.

**Figure 1:**
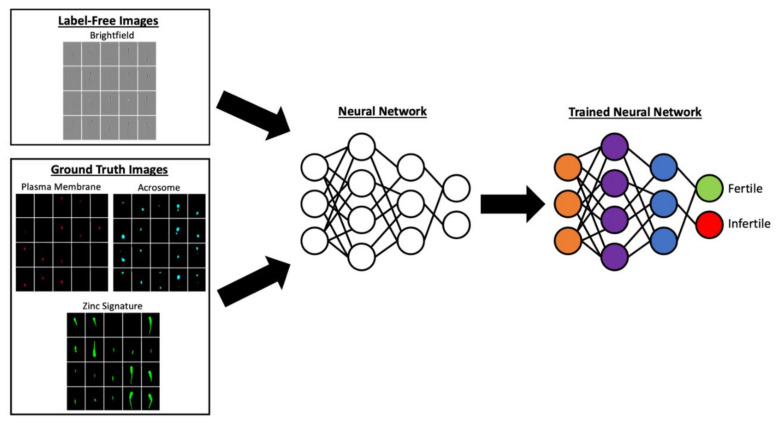
Biomarker sperm neural network training example. Images reflecting the biomarker status such as plasma membrane integrity (detected by propidium iodide), acrosome integrity (detected by lectin PNA conjugated to Cy5), and the sperm zinc signature (detected by FluoZin™-3 AM, ThermoFisher Scientific, Waltham, MA, USA) could be used to train neural networks to detect the percent of fertile and infertile sperm in a sample.

Furthermore, the potential for biomarker-based analysis is extensive as new sperm quality biomarkers continue to be identified [30,32,33,34,41,42]. New advancements to flow cytometry also allow for the dissemination of image-based flow cytometry (IBFC); this technology captures images of each single cell at high speeds (500 to 2000 sperm/second) and allows for characterizing biomarker localization at subcellular level (e.g., between sperm head and tail or within their respective sub-compartments), which is not possible in regular flow cytometry [43,44]. Coupled with artificial intelligence methods such as machine learning, IBFC in animal andrology may eventually lead to development of label-free approaches to semen analysis [45]. In this scenario, images from IBFC could have features extracted from ground truth images from biomarker characterization to train neural networks for differentiating fertile and infertile spermatozoa within an ejaculate based solely on bright field data with no need for sample labeling and fluorescence detection, an example is provided in Figure 1 [41]. Algorithms created by this type of research could then be utilized on bare bone image-based flow cytometers with no need of expensive lasers or on CASAs used in andrology labs daily.

## 4. Sperm Selection

The analysis of semen samples provides baseline data which is invaluable to the AI industry to improve the quality of sires and the semen they provide. The ability to use sperm quality parameters and biomarkers as targets to purify a semen sample is highly advantageous as well. By utilizing the same sperm quality parameters and sperm surface determinants previously mentioned, the quality of spermatozoa within each semen sample can be improved by rejecting defective spermatozoa and seminal debris. Sperm selection used in conjunction with more stringent sire and ejaculate selection will lead to an even greater increase in fertility. Additionally, the use of sperm selection methods can rescue a semen sample from a genetically valuable sire that produces substandard semen collections [32]; this application is the opposite of increasing male fertility at large but is still of economic value. Sires with valuable production traits but lower fertility will continue to be sought after; by purifying their semen collections, their AI fertility can be manually boosted.

Beyond the previously mentioned sperm sorting using biomarker-based flow cytometry, there are other promising sperm selection methods that are currently less laborious and require less expensive equipment. Colloid centrifugation is one such selection method. It selects spermatozoa based on motility, morphology, viability, and membrane integrity. The most common form of colloid centrifugation for sperm analysis is Single Layer Centrifugation (SLC) [46], which uses a single filter layer made from silica nanoparticles. SLC effectively purifies semen samples using a gentle centrifugation. Motile spermatozoa can line up in the direction of the centrifugation forces and pass through the silica layer. Additionally, those spermatozoa which are morphologically normal and have intact plasma membranes and chromatin pass through the colloid layer more easily [46]. This purification method has been studied in swine [47], sheep [48], cattle [49], and horses [50]. In all species, it was able to reliably improve the quality of the purified semen samples and in horses SLC has successfully increased AI conception rates in field trials by nearly 14% [50]. SLC purification can improve sperm quality in semen samples while remaining relatively cheap and simple to apply. Additionally, SLC selected sperm samples have maintained post-thaw viability better than non-selected samples when used in conjunction with semen freezing protocols [51,52]. This technique seems promising; however, field AI trials must still be conducted in other species. Furthermore, the method may not be conducive to boar semen purification due to large semen volume.

Nanopurification is another selection method which selects spermatozoa based on some of the same sperm quality biomarkers used in FC-based semen analysis. Specific probes can identify abnormal spermatozoa based on surface determinants (proteins, glycans) that relate to sperm quality [53]. Nanopurification uses these same negative biomarkers to remove abnormal or defective spermatozoa. Magnetic nanoparticles are coated with probes such as lectins, recombinant proteins and antibodies, that are known to bind negative biomarkers found on abnormal spermatozoa’s surface. These bound spermatozoa can then be removed from a sample using a magnet, without the need for sperm-damaging centrifugation or filtration [53]. The nanoparticles and the spermatozoa bound to them are drawn to the bottom of the tube by a magnet to form a plaque/pellet of bound, abnormal spermatozoa. The particle free spermatozoa are then pipetted out with the rest of the supernatant and ready for use in standard freezing, extending, or AI protocols [53]. Developed originally for bull spermatozoa and validate by AI field trials, the technique has been more recently adapted for boar [54,55] and even human semen purification [56].

This technique is quite simple once the coated nanoparticles are made. Nanoparticles are incubated with the semen samples for fifteen minutes at room temperature, and then, the sample is placed on a magnet for fifteen minutes. PNA-lectin is a probe which reliably binds spermatozoa with compromised acrosomes; a half-dose of semen purified with PNA-lectin coated nanoparticles was able to yield conception rates equal to a full dose of non-purified semen from the same bull. This demonstrates that the improvement of quality of semen acts in a compensatory manner for semen quantity. This technology has yielded calves that are appropriately developed and healthy [53]. Additionally, nanopurification has been experimentally implemented in swine utilizing negative biomarker probes for reacted acrosomes and apoptotic spermatozoa and was found to result in an equivalent fertility rate to non-selected spermatozoa. The resulting piglets developed normally and were healthy [54]. Nanopurification has encouraging outcomes and possibilities; it can easily be applied using other probes, which may be even more useful than PNA-lectin. Additionally, more than one type of probe can be used in a nanopurification at the same time, which would further purify the semen. Nanopurification is easily adaptable across species; it is a cheap and effective way to ameliorate sperm quality within a semen dose [53,54].

In summary, selection methods based on gradient separation and nanopurification are cheap, simple, and modifiable. Both methods can be species-optimized and further improved with better filters/gradients or probes. Furthermore, both methods have been linked to better AI fertility and semen quality [47,48,49,50,53,54,57]. Either method can be used to improve semen quality and potentially rescue semen samples from sub-fertile males. Though these selection methods are relatively new, they are promising and will be further optimized in the near future.

## 5. Sperm Sexing

A specialized form of semen selection which has been greatly improved upon in the past decade and continues to be optimized is sperm sexing. This is a method of selection which targets either X or Y chromosome bearing spermatozoa and effectively “purifies” a sample to contain a high percentage of spermatozoa with the desired sex chromosome. The most popular method of sexing and the method which has been utilized for commercial application is known as the Beltsville Sperm Sexing Technology. This technology was commercialized in 2004 by Sexing Technologies and has been adopted for use in the cattle industry. It is most substantially used by the dairy cattle industry, where a heifer calf is of far greater value than a bull calf, as females are necessary to produce milk, the intended product of the dairy industry.

Sex-sorted semen is sorted using a modified high-speed flow cytometer, which separate spermatozoa based on DNA content [58]. The flow cytometer measures DNA content based off the DNA-binding fluorescent probe Hoechst 33342. Spermatozoa containing the male, Y, chromosome lacking one arm have measurably less fluorescence than the spermatozoa containing X chromosomes, thereby allowing the flow cytometer to sort the sperm into X and Y groups based on the amount of fluorescence that the spermatozoa emit when excited by a laser within the flow cytometer [58]. According to Sexing Technologies, they are able to sex cattle semen with 93% gender accuracy. Though sexed semen has been available for cattle producers for over a decade, this product is still in its infancy across all other livestock species. The first goat kids were born using sexed semen in 2013 [15]. As previously mentioned, the first small ruminant sexed semen company was launched as of August 2020, by Animal Breeding Europe and Cogent. The swine industry experimented with sexed semen earlier and produced the first sexed semen piglets in 1998 [17]. As previously mentioned, Sexing Technologies is actively attempting to commercialized sex boar semen; however, no swine-sexed semen products are available for routine use at this time [21]. Additionally, the cost of sexed semen is higher than that of conventional semen. Doses of sexed cattle semen cost anywhere from USD 15 to USD 30 US more per dose [59]. This increase in cost will be seen in sexed semen products for the other livestock species as well. Though sexed semen is only routinely used in cattle right now, this area of the industry is continuing to grow; the efficient sexing of spermatozoa in all species is of great interest for the livestock industry.

Currently, cattle-sexed semen has a fertility rate of 71.5% to 78.5% that of whole semen [60]. Though these reduced rates are acceptable for dairy producers and some beef producers to use sexed semen, fertility rates are still the foremost issue facing sexed semen. Part of the issue is the large amount of handling the sexed sperm undergoes, as well as high pressure, speed and shear force, and speed of sheath fluid carrying through the cell sorter. The sexing process involves nearly 30 steps of processing, and the spermatozoa are exposed to several different media through-out the processing [14]. Furthermore, the actual sexing, though becoming faster all the time, is still quite slow (100 to 200 million spermatozoa/hour). Increased handling and slow processing reduce the life span of spermatozoa and can result in damaged spermatozoa [61,62], which makes the stable storage of the spermatozoa more difficult.

The bull sire company ABS Global, a subsidiary of Genus plc, produces their sexed semen product Sexcel using another form of sperm sexing technology: gender ablation sperm sexing. This technology uses machinery similar to Beltsville Sperm Sexing. Spermatozoa DNA is stained with Hoechst 33342 and categorized as an X or Y containing spermatozoa. The key difference in this method is that the undesired gendered spermatozoa are then destroyed using a specialized laser rather than sorted into different X and Y collection tubes using a cell sorter. The result is a collection skewed only to the desired gender of spermatozoa, rather than two separate collections with one skewed to Y and the other skewed to X, as is the end product of the Beltsville Sperm Sexing technique. Gender ablation sperm sexing resulted in a conception rate which was 78% that of conventional semen in a recent beef cattle AI field trial [63]. However, ABS Global’s website indicates that in dairy operations Sexcel has achieved a 90% relative conception rate when compared with conventional semen [64]. These results are similar to or may even surpass sexed semen results from Beltsville Sexing Technology. Many challenges remain in this sector of the industry, the greatest of which appears to be safe spermatozoa processing during sexing, a problem that persists in both Beltsville Sexing and Gender ablation sexing.

In response to the issues of overhandling of spermatozoa, an alternative sexing method has been explored. This method is nanopurification, as previously discussed, but with the use of a probe that can presumably detect differences on the surface of X and Y spermatozoa. Nanoparticle sexing would be highly effective if a reliable probe can be found and utilized. Currently the only nanoprobe which has been used successfully is nanoparticles coated in silica, which contains a negative charge and thus can interact with the Z-electrical potential of the sperm membrane, which is measurably different between X (−20 mV) and Y (−16 mV) spermatozoa. Because of the difference in charge, Y spermatozoa remained closer to the nanoparticles and could be magnetically attracted to the bottom of the semen sample and the X containing spermatozoa could be removed with the supernatant. This charge-based nanopurification method sex-sorted donkey spermatozoa with 90% accuracy [65]. Thus far, this technique has only been attempted in donkeys but has promising preliminary results. Furthermore, this method has yet to be tested by field AI trials which will be a key step in further developing this technology. The discovery of other sex-specific spermatozoa differences could also be utilized via this nanopurification technique, adding the benefit of defective sperm removal to that of sex selection.

Another alternative sexing method which would require less sperm handling is immunological sperm sexing. Similar to nanopurification, this method utilizes surface differences between Y and X spermatozoa. Specifically, one antigen known as histocompatibility-Y antigen (H-Y antigen), which is found on the majority of Y bearing spermatozoa, but found on only a small percentage of X bearing spermatozoa [66]. Antibodies for the H-Y antigen have been developed in mouse and can be used to induce Y sperm cytotoxicity as a method of sexing [67]. This cytotoxicity results in the death of the targeted spermatozoa, in this case those that carry the Y chromosome. In cattle, when compared with conventional semen, immunological sexed semen showed no difference in acrosomal integrity but did have a higher percentage of head and tail defects. However, in an AI field trial comparing the two methods, immunological sorting produced a significantly higher percentage of female calves (74.29%) than conventional semen (47.22%), with no difference in pregnancy rates between the two methods [68]. Immunological sorting is also cheaper than using a flow cytometer for sperm sexing, and produces a higher number of sperm per dose [68]. This sexing method requires further refinement however it is promising. The most reassuring aspect of immunological sexing is the equivalent pregnancy rates between conventional semen and immunologically sexed semen. A previously mentioned and notorious critique of the Beltsville Sperm Sexing flow cytometry-based sperm sexing method is the reduced pregnancy rates associated with it. Immunological sexing, though not as gender accurate, experiences no reduction in pregnancy rates. Through further refinement this sexing system will hopefully increase in its gender accuracy without experiencing a decrease in pregnancy rate, an outcome that would be quite valuable in the cattle industry. Finally, the use of H-Y antigen with the previously described nanopurification method of sexing would be an interesting collaboration which could perhaps combine these two methods of sexing and ultimately produce an even better outcome.

Sperm sexing is and will remain one of the fastest growing technologies within the AI sector. The value of sexed semen has already been demonstrated by dairy producers and much work is being carried out to implement this technology in other livestock species, also. Regardless of the method, improvements in sexing will allow producers to manage their herd gender dynamics and add profitability by producing the gender of offspring they desire for their operation. Improved sexed semen outcomes will likely raise the adoption of AI in beef cattle, horses, and small ruminants because being able to select the gender of offspring from each individual sire can be such a huge advantage for a producer. Furthermore, the dissemination of sexed semen in the swine industry would be quite advantageous, as previously discussed. All these sexing technologies are highly inventive and are showing how powerful precision agriculture can be in real world applications. Undoubtedly, these technologies will be further refined and improved. The ability to reliably sex spermatozoa is hugely advantageous in the livestock industry and gives producers greater control over their herd dynamics.

## 6. Semen Storage

Liquid extenders are currently the most popular method for storing semen in all the livestock species besides cattle. Though liquid extenders are widely used, they leave much to be desired. Boar extenders have been a subject of extensive research to improve storage efficiency [13]. Both equine and small ruminant extenders are currently lagging behind and have a shorter shelf life than extended boar semen [11]. Many different extender supplementations have been attempted and have resulted in improvements [13]; however, the shelf life of liquid extended semen is still much shorter than frozen, or cryopreserved, semen. There is considerable effort being made to fine-tune the efficiency of freezing semen for all species. Though there are currently protocols that attempt to maximize the use of liquid stored sperm, successful semen cryopreservation protocols are a priority for all livestock species [12].

Liquid extenders include antibiotics, as oftentimes the storage environment (e.g., temperature, energy substrates, pH, etc.) support bacterial growth. Antibiotic resistance is an increasing concern. To date, there are primarily two ways around this. One is to make use of hypothermic storage as alluded to in the Horse section. In boar semen storage, hypothermic storage consists of storing extended semen at 4–5 °C [23,69]. This brings about added stress to sperm cells and the inclusion of additional compounds that allow the cells to survive at this temperature. Storage at this temperature helps decrease the ability of bacteria to multiply. Alternatively, others have suggested use of bacteriostatic agents in the plastic of dose containers. These bacteriostatic agents, in practice, do not kill the bacteria, but rather prevent them from multiplying. Follow up studies should compare these two methods.

Cryopreservation is more effective for the long-term storage of spermatozoa than liquid extender storage [70]. The main hurdle for cryopreservation of semen is the retention of acceptable viability and fertility from the semen dose upon thawing [13]. Cattle semen can be cryopreserved effectively using a standardized slow-freezing protocol [71]. This is not the case with other species. Boar spermatozoa in particular are very susceptible to cold shock [70], and as a result, the spermatozoa that undergo freezing are often weakened [72]. These weakened spermatozoa can suffer from DNA fragmentation, degradation of proteins and RNAs contained within the spermatozoa, disruption of the acrosome and sperm membranes, as well as reduction in mitochondrial activity and sperm motility [70]. All these issues can result in decreased fertility from a semen dose [72].

Freezing semen is further complicated by the variable tolerance to freezing between different sires and even different collections from the same sire [12]. This variability has prompted researchers to explore the possibility of biomarkers which can determine the ability of a particular sire or ejaculate to be frozen [73]. If such biomarkers were identified and used in conjunction with FC-based semen analysis and nanopurification, it would be feasible to devise a method to analyze and purify semen for maximum freezing potential, and even select sires with highest sperm cryotolerance. Freezability biomarkers have been explored in bulls, where higher expression of voltage-dependent anion-selective channel protein 2 (VDAC2) and glutathione S-transferase mu 5 (GSTM5) in spermatozoa negatively correlated with freezability, and higher levels of ATPase synthase subunit beta (ATP1B1) were associated with better freezability [74]. Similar findings were observed in boars, where a higher expression of VDAC2 in spermatozoa was also associated with better freezability [75], and a higher expression of glutathione S-transferase mu 3 (GSTM3) in spermatozoa was also associated with a lower freezability in boar ejaculates [76]. Other freezability biomarkers which have been identified in boar are acrosin binding protein (ACRBP) which was found to be significantly lower in poor freezing ejaculates and triosephosphate isomerase (TPI) which was found to be significantly increased in poor freezing ejaculates [77]. Furthermore, there are freezability biomarkers detectable in seminal plasma as well. In bulls, higher expression of heat-shock proteins in the seminal plasma was associated with cryotolerance [78,79]. Additionally, the presence of other proteins in seminal plasma has been associated with higher or lower cryotolerance. The presence of lipocalin-type prostaglandin D synthase (L-PGDS) has been associated with poor freezing [80], whereas the expression of an acidic seminal fluid protein (aSFP) has been associated with better freezing and seems to protect spermatozoa during the freezing process [81]. In boars, one of the most reliable freezability markers is the presence of fibronectin-1 (FN-1) in boar seminal plasma, an increase in FN-1 is associated with increased freezability [70]. There also exists evidence that freezability itself is a genetically heritable trait [82]. Some of the aforementioned biomarkers and their differing presence in specific males can almost certainly be explained by differing genetics between males [82,83,84].

Correlating freezability itself and these biomarkers to specific genes may help researchers further grasp the intricacies between variable tolerances to sperm freezing in males. Additionally, the practice of diagnosing semen samples’ cryotolerance and assessing the level of cryodamage after thawing will improve the outcomes of freezing in the non-cattle livestock species. Altogether, these approaches will further improve frozen semen within cattle AI also.

Sperm lyophilization/freeze-drying has been considered as an alternative storage method based on live births with lyophilized mouse and rabbit spermatozoa following the transfer of embryos conceived by intracytoplasmic sperm injection (ICSI) [85,86]. However, this approach may not be suitable for commercial embryo transfer in livestock species because of the high equipment and labor cost of ICSI. Furthermore, lyophilization is likely to cause sperm-borne centriole damage in non-rodent species (rodent spermatozoa do not carry this otherwise essential microtubule organizing organelle), and sperm DNA damage by lyophilization is a concern in all species [87]. Although some progress has been made toward understanding the centrosomal inheritance issue after sperm lyophilization in livestock species [88,89], DNA damage and poor developmental potential after freeze-dried ICSI are yet to be addressed.

Both liquid extenders and frozen semen protocols can benefit from improvements in analysis and selection technologies. As previously stated, much of the work carried out in this field of study revolves around the ingredients used within the extenders [13]; however, focusing on the spermatozoa which are actually extended or frozen is of importance. The previously discussed analysis and selection methods will produce semen samples with more robust spermatozoa capable of withstanding extension and freezing protocols better. Such results have already been shown using SLC selection protocols [51,52]. Improving semen storage will greatly benefit the AI industry and using more effective sperm analysis and selection methods will improve semen storage outcomes. Additional advances in frozen semen storage and distribution could stem from ongoing research on sperm cryoprotectant improvement and sperm preservation at temperatures above the temperature of liquid nitrogen-dependent sperm storage [90,91].

## 7. Conclusions

The AI industry continues to see growth in value and popularity [7]. There are many ways in which AI can be improved but increasing AI fertility rates and semen storage are of utmost importance. Though the popularity of AI varies greatly between livestock species, all the species experience issues with varying male fertility rates [29]. The adoption of more stringent semen analysis procedures such as CASA and FC-based analysis can effectively eliminate sub-fertile males. Furthermore, the use of sperm selection methods can improve AI dose quality from sub-fertile males which have premier genetics related to production or competition, in the case of stallions. Sperm sexing has seen a dramatic rise in popularity since it was first commercialized in 2004 and is an AI technology which continues to grow and improve. Effective sperm sexing will almost certainly drive a greater adoption of AI use and has huge financial and logistic implications for producers. Storage of semen is also an issue within all the species and the use of effective sperm selection protocols will result in more robust spermatozoa for storage and thus improve this aspect of the industry as well. Though many of these practices and improvements can be applied across species, it is important to remember that each species has its own nuances as far as semen production and sperm analysis is concerned. Though many of the methods for improving AI are similar between species, it is important to remember that each species has its own unique differences in regard to sperm characteristics and also management practices within that sector of the industry. The nature of each industry has nuances for the end user of these semen products and researchers must keep these in mind when attempting to improve semen quality. Ultimately, the adoption of high precision, biomarker-based semen analysis, sperm selection, sperm sexing, and semen storage methods such as those discussed in this paper will further boost the usefulness of AI and improve semen products for AI across the industry.

## Data Availability

Not applicable.
